# Does the provision of live black soldier fly and yellow mealworm larvae improve Muscovy duck welfare?

**DOI:** 10.1186/s40104-023-00949-7

**Published:** 2023-12-04

**Authors:** Marta Gariglio, Sihem Dabbou, Manuela Renna, Ilaria Biasato, Sara Bellezza Oddon, Marco Meneguz, Raul Daniel Miazzo, Stefania Bergagna, Elena Colombino, Elisabetta Macchi, Achille Schiavone

**Affiliations:** 1 Department of Veterinary Sciences, University of Turin, Largo Paolo Braccini 2, Grugliasco, Torino (TO), 10095 Italy; 2 Center Agriculture Food Environment (C3A), University of Trento, Via Edmund Mach 1, San Michele All’Adige, Trento (TN), 38098 Italy; 3 Department of Agricultural, Forest and Food Sciences, University of Turin, Largo Paolo Braccini 2, Grugliasco, Torino (TO), 10095 Italy; 4 BEF Biosystems s.r.l, Via Tancredi Canonico 18/C, Torino (TO), 10156 Italy; 5 Unidad de Investigación Aviar, Producción Avícola, Fac. Agr. y Vet., Universidad Nacional de Río Cuarto, Cordoba, Argentina; 6 Veterinary Medical Research Institute for Piemonte, Liguria and Valle d’Aosta, Via Bologna 148, Torino (TO), 10154 Italy

**Keywords:** Behavior, *Cairina moschata*, Environmental enrichment, *Hermetia illucens*, Poultry, *Tenebrio molitor*, Welfare

## Abstract

**Background:**

The provision of environmental enrichments to Muscovy ducks could reduce the expression of the aggressive behaviors. The aim of the present study was to evaluate the effects of black soldier fly (BSF) and yellow mealworm (YM) live larva provision on Muscovy duck performance, excreta corticosterone metabolites (ECM), behavior, and blood parameters.

**Methods:**

A total of 126 3-day-old female Muscovy ducklings were allotted to 18 pens (6 replicates/treatment, 7 birds/pen) and assigned to 3 experimental treatments: a control group fed commercial feed, and two experimental treatments fed commercial feed plus the 5% (based on the expected daily feed intake, as fed basis) of BSF and YM live larvae (BSF and YM groups, respectively). A two-phase feeding program was applied: starter (from 3 to 31 days of age) and grower-finisher (from 32 to 55 days of age). The live weight, average daily gain, average daily feed intake, and feed conversion ratio were calculated. Larva consumption times were collected, and video recordings were performed during 3 periods (P) each day: the hour before (P1), during (P2), and after (P3) the larva administration. ECM were evaluated at 3, 31, and 55-day-old. Finally, the total red and white blood cell counts, serum proteins, lipids, and liver and renal function serum enzymes were evaluated on 12 birds/treatment.

**Results:**

The experimental treatment did not affect the growth performance of the birds (*P* > 0.05). Larva consumption times were always similar between the two insect species, except at 14–18 days of age, were BSF larvae were consumed faster than YM larvae (*P* < 0.001). The birds showed less walking activity during P2, and preening behavior increased in YM birds during P3. The C birds increased the attack behavior over the weeks (*P* < 0.05). During weeks 1–3 the YM group reduced the attack frequency (P1 > P3; *P* < 0.05). Finally, the provision of live BSF and YM larvae significantly reduced the ECM at 55 days of age and the heterophil to lymphocyte ratio (*P* < 0.05).

**Conclusions:**

Live BSF and YM larva supplementation in Muscovy duck improves duck welfare, without impairing birds’ growth performance.

**Supplementary Information:**

The online version contains supplementary material available at 10.1186/s40104-023-00949-7.

## Background

Assessments of animal welfare have increased significantly over recent years due to consumers’ increasing interest in the living conditions of farmed animals [1, 2]. Indeed, 34% of a sample of European citizens reported being concerned about animal welfare in 2006, rising to 57% in 2016 [3]. From a survey carried out on European consumers, different aspects of animal welfare were highlighted as being important [4]. First, consumers indicated “space allowance” as the welfare aspect they were most concerned about, followed by the possibility for animals to have access to outdoor areas, the absence of movement restrictions, and the possibility for animals to perform their natural behaviors [5]. The evaluation of animal welfare requires an interdisciplinary approach, since it comprises good health, comfort, and the expression of natural behaviors by the animals [1, 6].

Corticosterone is an indicator of stress status, being produced by the hypothalamus-pituitary-adrenocortical cascade in the case of stressful events or situations (for example, fear of humans, fear of capture, and handling-related stress) [7]. Of the different existing techniques, excreta corticosterone metabolites (ECM) determination could be chosen due to its non-invasive nature and feedback-free approach [8, 9]. On the other hand, the heterophil to lymphocyte ratio (H/L) is well known to be a hematological stress indicator, being considered a valuable tool in the determination of the health status of poultry [10, 11].

In farmed animals, welfare issues mainly derive from the inability to express natural behaviors, that can evolve into frustration, abnormal behaviors, and injuries [12]. Regarding domestic ducks, their natural behavioral pattern is very similar to that of their progenitor, being efficient walkers, flyers, and swimmers [12, 13]. Moreover, ducks naturally spend a lot of time performing preening behaviors, including body shaking and cleaning [13]. In the case of confinement rearing, high stocking densities, and/or the absence of litter, a duck may start to express abnormal behaviors, that could damage the bird itself and/or its conspecifics [12]. The main behavioral problems in ducks reared for meat production are injurious feather pecking and cannibalism [12, 13]. Providing the ducks with a more stimulating environment could help to reduce aggressive phenomena, for example, by providing a water bath, an enriched feed supply (distributed in form of straw or hay), or outdoor access to the birds [12, 14]. Indeed, the provision of an outdoor space with bathing water supplementation as well as low stocking densities (6.3 vs. 11.6 birds/m^2^) were shown to reduce feather pecking significantly in Muscovy ducks (*Cairina moschata domestica*) [15, 16].

Recently, the effects of providing live insect larvae of the black soldier fly (BSF, *Hermetia illucens*) and yellow mealworm (YM, *Tenebrio molitor*) as environmental enrichment in poultry has been examined by a number of authors, who have evaluated their effects in terms of growth performance and animal behavior [17–19]. The results on growth performance vary greatly between the studies, probably due to the high variability in terms of the amounts of larvae provided and the methods adopted for larva provision (provided as a supplementation of the diet, included in the diet formula or scattered throughout the pen) and the avian species considered. In a study conducted by Bellezza Oddon et al. [18], BSF and YM live larva provision [5% of the expected average daily feed intake (ADFI) once a day] to broiler chickens resulted in improvements in birds’ growth performance, with the YM-supplemented birds showing a better feed conversion ratio (FCR) compared to the control group. The study by Ipema et al. [19], on the other hand, studied the administration of two different levels of live BSF larvae [5% and 10% of the estimated daily dry matter (DM) intake, twice a day] to broiler chickens, and found that the final live weight (LW) of the animals fed 10% DM live larvae was lower than that of those fed 5% DM live larvae or the control diet [19]. By contrast, body weight gain and the FCR were both improved in turkeys by the daily administration of live BSF larvae (10% of the expected ADFI, once a day) [20]. From a nutritional point of view, it is well known that insects are a nutrient-rich feedstuffs for livestock animals, being a good source of protein (with high biological value in terms of amino acid content), fat and energy. However, considering the application of whole insects as environmental enrichment, their water content (around 70%) must be considered, since it highly reduces the amount of provided nutrients (on a DM basis) [17]. On the other hand, from the results obtained in previous studies, it can be supposed that larvae motility might affect their attractiveness to the birds and encourage foraging behavior and the birds’ activity in general [19–21]. However, also in this case, the results vary consistently depending on the approach adopted in terms of the quantity of larvae and the modality of their provision. Investigating different amounts and frequency (5% vs. 10%, 2 or 4 times/d) of BSF live larva provision in broilers, Ipema et al. [19] observed more active behaviors in insect-fed groups compared with control birds; moreover, more active behavior was observed the greater the amount and frequency of live larva administration. In another study, BSF and YM live larva provision in broilers was associated with higher levels of ground pecking and scratching [22]. Similarly, foraging-related behaviors tended to be higher in turkeys fed insects (10% of the expected ADFI) compared with the control group [20].

Like wild ducks, Muscovy ducks are omnivorous, feeding on a variety of different vegetable and animal sources, and they are good insect hunters [13]. Thus, the dietary supplementation of live insect larvae in Muscovy ducks could be a useful tool to improve their living environment, and thus the welfare of birds raised in captive farming conditions, being a positive enriching stimulus, reduce the expression of aggressive phenomena and abnormal behaviors.

For this reason, the aim of the present study was to evaluate the effects of BSF and YM live larva provision in intensive farming systems on Muscovy duck performance and welfare status, evaluated in terms of ECM, duck behavior, and blood parameters.

## Materials and methods

### Birds, husbandry, and feeding

A total of 126 3-day-old female Muscovy ducklings (Canedins R61 Barred blue, Grimaud Freres Selection, France) were used. The trial was conducted at the poultry experimental facility of the Department of Agricultural, Forest and Food Science of the University of Turin (Italy). The poultry house was 7 m wide × 50 m long × 7 m high, with an automatic ventilation system and equipped with a waterproof floor and walls. Each pen measured 1.20 m × 2.20 m, and rice hulls were used as bedding material. Infrared lamps were used to heat the birds during the first three weeks of life. In the first 3 d of the trial (up to a bird age of 6 d), the daily lighting schedule was 23L:1D; then, the dark period was progressively increased up to 6 h and then maintained constant until the end of the trial. The environmental parameters, mortality rate and clinical signs of illness were monitored daily during the whole trial.

Upon their arrival at the experimental facility, each bird was individually labeled with a wing mark and then randomly distributed between the 18 pens (6 replicates/treatment, 7 birds/pen), with an average LW of 80.3 ± 7.28 g (for all ducks, mean ± standard deviation). The three experimental treatments were as follows: the control group (C), fed with commercial feed; the BSF group, fed with commercial feed + 5% supplemented live BSF larvae (on an as fed basis); the YM group, fed with commercial feed + 5% supplemented live YM larvae (on an as fed basis). A commercial based diet was used for all the experimental treatments, provided by Borello Mangimi S.r.l. (Bra, CN, Italy), and a 2-phase feeding program was applied: starter diet (from 3 to 31 days of age; apparent metabolizable energy corrected for nitrogen [AMEn]: 2,696.1 kcal/kg, as fed; crude protein [CP]: 19.3%DM) and grower-finisher diet (from 32 to 55 days of age; AMEn: 2,741.9 kcal/kg, as fed; CP: 17.9%DM) (Table 1). Considering the ADFI (as fed basis) reported on the previous study on Muscovy ducks by Gariglio et al. [23], the amount of live insect larvae to be provided to ducks was calculated, accordingly to the 5% of the expected ADFI (as fed basis).

**Table 1:** Ingredients (g/kg as fed) and nutrient composition (g/100 g DM, unless otherwise stated) of the experimental diets

**Ingredients**	**Starter period (d 3 to 31)**	**Grower-finisher period (d 32 to 55)**
Corn meal	418	541
Soybean meal	292	234
Bran meal	53.4	60.0
Common wheat	150	57.8
Wheat meal	34.4	50.0
Soybean oil	10.0	12.0
Calcium carbonate	15.7	22.9
Dicalcium phosphate	12.3	9.90
Sodium bicarbonate	2.50	2.10
Sodium chloride	2.00	1.90
DL-methionine	2.50	1.80
L-lysine HCl	0.900	1.70
Mineral-vitamin premix^a^	4.00	3.00
Optifos 250 bro^b^	1.00	1.00
Avizyme 1500X^c^	1.00	1.00
Total	1,000	1,000
AMEn, kcal/kg (as fed)	2,696	2,742
Analyzed nutrient composition		
Dry matter, g/100 g	90.9	90.6
Crude protein, g/100 g DM	19.3	17.9
Ether extract, g/100 g DM	2.51	3.23
Ash, g/100 g DM	6.50	6.52
Amino acids, g/100 g on (as fed basis)		
Lysine	1.01	0.932
Methionine	0.514	0.426

*AMEn* Apparent metabolizable energy corrected with nitrogen retention, *DM* Dry matter

^a^Mineral-vitamin premix per kg: vitamin A (retinyl acetate), 12,500 IU; vitamin D_3_ (cholecalciferol), 3,500 IU; vitamin E (DL-α-tocopheryl acetate), 40 mg; vitamin K (menadione sodium bisulfite), 2.0 mg; biotin, 0.20 mg; thiamine, 2.0 mg; riboflavin, 6.0 mg; pantothenate, 15.21 mg; niacin, 40.0 mg; choline, 750.0 mg pyridoxine, 4.0 mg; folic acid, 0.75 mg; vitamin B_12_, 0.03 mg; Mn, 70 mg; Zn, 62.15 mg; Fe, 50.0 mg; Cu, 7.0 mg; I, 0.25 mg; Se, 0.25 mg

^b^Optifos 250 bro: Phytase (EC 3.1.3.26) (250 OTU/kg diet), Huvepharma, Sofia, Bulgaria

^c^Avizyme 1500X: Complex of Endo 1–4-Beta-Xylanase (EC 3.2.1.8) (256 U/kg diet), Subtilisin (EC 3.4.21.62) (2,560 U/kg diet) and α-Amylase (EC 3.2.1.1) (1,472 U/kg diet), Danisco Animal Nutrition, Marlborough, Wiltshire, UK

The amounts of live BSF and YM larvae to be supplied in each pen were weighed daily and then administered in a feeding plate. The live insect larvae were given daily at 10:00 h. The C group received an empty feeding plate at the same time, to ensure that interactions between the operator and the birds were the same for all the groups.

Following the protocol provided by Bellezza Oddon et al. [18], two different dimensions of insect larvae were provided for both BSF and YM, according to the age and size of the birds: early (0.8 ± 0.05 cm) or late instar stage larvae (1.5 ± 0.05 cm) were provided during the starter and grower-finisher periods, respectively.

The live BSF larvae were obtained from the experimental facility of the Department of Agricultural, Forest and Food Science of the University of Turin (Italy), while the live YM larvae were obtained from Italian Cricket Farm (Scalenghe, Turin, Italy). Both the BSF and YM larvae were reared on vegetable substrates (Gainesville diet—based on corn meal, wheat bran, alfalfa—for BSF larvae and mainly wheat bran for YM larvae). Once per week the new batches of both live BSF and YM larvae were stored in cool rooms according to the method reported by Bellezza Oddon et al. [18].

### Chemical analyses

Chemical analyses were carried out on the commercial diets and on pooled batches of larvae. The diet samples were ground to pass through a 0.5-mm sieve and stored in airtight plastic containers until analyses. A sample of each batch of larvae was stored (−20 °C), freeze dried, ground and analyzed for DM (method number 943.01), CP (method number 984.13), ether extract (EE, method number 2003.05), and ash (method number 924.05), according to AOAC International [24, 25]. The gross energy (GE) content of the larvae was determined using an adiabatic calorimetric bomb (C7000; IKA, Staufen, Germany). Finally, the amino acid analysis was performed as reported by Bongiorno et al. [17]. The proximate composition of the BSF and YM larvae of both early and late instars is reported in Table 2.

**Table 2:** Proximate composition (g/100 g as fed) of BSF and YM live larvae provided to Muscovy ducks

**Item**	**BSF early instar**	**BSF late instar**	**YM early instar**	**YM late instar**
DM	25.3	24.8	38.3	37.6
CP	10.4	11.2	18.3	14.7
EE	5.01	6.13	1.50	1.82
Ash	2.02	2.63	2.51	2.33
GE, MJ/kg	5.25	6.63	9.31	10.4
Amino acids, g/100 g (as fed basis)				
Lysine	0.274	0.272	0.895	1.00
Methionine	0.803	0.705	0.981	0.594

*BSF* Black soldier fly, *YM* Yellow mealworm, *DM* Dry matter, *CP* Crude protein, *EE* Ether extract, *GE* Gross energy

### Growth performance

The birds were weighed at the beginning of the trial and at the end of the two feeding phases: 3 days of age, 31 days of age (end of starter period), and 55 days of age (end of the growing period and of the trial). The average daily gain (ADG), ADFI, and FCR were calculated at the pen level at the end of each feeding phase and for the overall experimental trial. The ADFI was calculated on the as fed basis considering the average amount of larvae provided. Finally, the DM of the live BSF and YM larvae (25.26 and 38.30 g/100 g for BSF and YM early instar, 24.85 and 37.56 g/100 g for BSF and YM late instars, respectively) was used to adjust the FCR, according to the method reported by Bongiorno et al. [17]. All the measurements were made using an electronic scale (Sartorius – Signum®, Bovenden, Germany; d = 0.1).

### Larva consumption time and behavioral observations

After an adaptation period of 3 d to allow the ducks to become accustomed to the consumption of larvae, the larva consumption times for each pen were recorded by stopwatch, for 5 d per week from the d 4 of trial (7 days of age) until the end of the experiment. For this evaluation, the operator stood in front of the pen and the stopwatch was started when the plate was inserted into the pen, and stopped when the supplied larvae were completely consumed by the birds. When the larvae were completely consumed by the birds, the plates were immediately removed from each pen. To evaluate how the larva consumption time varied on a weekly basis, the daily data were then regrouped in intervals of 5 d (7–11, 14–18, 21–25, 28–32, 35–39, 42–46, 49–53 days of age).

Behavioral observations were made using video recordings. Video recordings were taken on the same 3 replicates/treatment (randomly selected), 1 d/week, starting from the second week of the trial until the end of the experiment. The video recordings were made during 3 periods (P) of the day as follows: the hour before insect larva administration (P1, from 9:00 to 10:00 h), the hour during the larva provision (P2, from 10:00 to 11:00 h, concurrently with larva consumption time recording), and the hour after insect larva administration (P3, from 11:00 to 12:00 h). For each hour, the behavior of all the ducks in the pen was recorded and observed, according to the ethogram reported in Table 3 (adapted from Veldkamp and van Nieken [20], Riber and Mench [26], Jones and Dawkins [27], O’Driscoll and Broom [28]). Data recorded during the observations were subsequently analyzed with BORIS (Behavioral Observation Research Interactive Software) version 7.13.8 [29]. The observed behaviors were divided between state and point events: state events were those that were expressed over a length of time, whereas point events were all those that were brief and sudden, and so did not involve a length of time (Table 3). State events were then reported as the mean time of the specific behavior expressed for the hour of observation, while the point events were expressed as a frequency for the hour of observation. To evaluate the effect of the age of the birds on their behavioral repertoire, the behavioral observation data were then grouped according to the feeding periods: weeks 1–3 (W1–3) for the starter period and weeks 4–6 (W4–6) for the grower-finisher period.

**Table 3:** Ethogram of activity, foraging, feather caring and aggressive behaviors (adapted from Veldkamp and van Nieken [20], Riber and Mench [26], Jones and Dawkins [27], O’Driscoll and Broom [28])

**Behaviour category**	**Behaviour**	**Event**	**Description**
Activity	Rest	State	Laying down sleeping or without activity
	Stand	State	Standing without activity
	Walk	State	Walking, running, or trotting
Foraging	Eat	State	Pecking the feeder (other than insect tray)
	Drink	State	Pecking the bell drinker
	Eat insects	State	Pecking the insects
	Peck object	State	Pecking objects other than feed or water
Feather caring	Preen	State	Arranging feathers with the beak
	Shake	Point	Rapid shaking of the head, body, or tail
	Stretch	Point	Stretching movements of neck, wings, or legs
	Flap wings	Point	Beating the air with the wings
Aggressive	Attack	Point	Pecking a conspecific

### Excreta corticosterone metabolites

ECM levels were determined at a pen level at the beginning of the trial (T0, 3 days of age), at the end of the starter period (T1, 31 days of age) and at the end (T2, 55 days of age) of the trial (in different day than the video recordings). For the excreta collection, the birds of each pen were housed in wire-mesh cages (100 cm × 50 cm × 50 cm) for 60 min. After collection, the excreta samples were pooled and frozen at −20 °C. The ECM levels were determined according to Palme et al. [30] and Costa et al. [31]. The freeze-dried excreta were ground using a cutting mill (MLI 204; Bühler AG, Uzwil, Switzerland). Then, 0.25 g of each sample were placed into an extraction tube with 3 mL of ether and stored at −20 °C for 1 h. Then, the aliquots were mixed for 3 min using a multivortex (ArgoLab Mix, Giorgio Bormac S.r.l., Carpi—Modena, Italy), and the supernatant was collected and transferred in a new tube. A dried extract of the samples was obtained by drying the samples at 50 °C for 14 h. Finally, ECM were analyzed using a Corticosterone Multi Format Elisa kit (Arbor Assay®, Ann Arbor, MI, USA) validated for multiple biological substrates including fecal extracts. All the analyses were performed in duplicate. The inter- and intra-assay coefficients of variation were less than 10% (8% and 9%, respectively). The sensitivity of the assay was 10.5 ng/g of excreta. All the samples were analyzed at multiple dilutions (1:4, 1:8, 1:16 and 1:32) and all the regression slopes were parallel to the standard curve (*R*^2^ = 0.989).

### Blood analyses

At the end of the trial (55 days of age) all the birds were weighed, then 12 ducks per treatment (2 birds/pen) were selected based on each pen average LW, and blood samples were then collected from the jugular vein of the selected birds during the process of slaughtering. A 2.5 mL aliquot of blood was placed in an EDTA tube, and another was placed in a serum-separating tube. A blood smear was prepared from a single droplet without any anticoagulant. The total red (erythrocytes) and white (leukocytes) cell counts were determined in an improved Neubauer hemocytometer after mixing with a Natt‐Herrick solution at a 1:200 ratio as reported by Natt and Herrick [32]. The blood smears were stained with May‐Grünwald and Giemsa–Romanowski stains. One hundred white blood cells were evaluated per smear to determine the H/L, while the number of blood cell types was determined according to Campbell [33].

The serum-separating tubes were left in a standing position at room temperature for approximately 2 h until the formation of a blood clot. Then, the tubes were centrifuged at 700 × *g* for 15 min and the obtained serum was immediately frozen at −80 °C. Total protein content was quantified using the “biuret method” (Bio Group Medical System kit; Bio Group Medical System, Talamello (RN), Italy), and the electrophoretic pattern of the serum was assessed using a semi-automated agarose gel electrophoresis system (Sebia Hydrasys®, Norcross, GA, USA). Alanine-aminotransferase (ALT), aspartate-aminotransferase (AST), gamma glutamyl transferase (GGT), triglycerides, cholesterol, uric acid, and creatinine serum concentrations were measured using enzymatic methods in a clinical chemistry analyzer (Screen Master Touch, Hospitex diagnostics S.r.l., Firenze, Italy), as reported by Salamano et al. [34].

### Statistical analyses

Statistical analyses were performed using the SPSS software package (version 21 for Windows, SPSS Inc., Chicago, IL, USA). The mortality rate was analyzed by means of a Chi-square test, using the C group as the reference. The experimental unit was the pen for all the analyzed data. One-way ANOVA was used to analyze the collected data for growth performance, ECM, blood, and serum enzymes, using the Brown–Forsythe test to establish the normality or non-normality of the distribution. Moreover, for the larva consumption time, a general linear mixed model (GLMM) with a gamma probability distribution and log-link function was used to compare the larva intake-time in seconds as a function of two fixed factors (treatment and day of age) and their interaction. The replicate was included as a random effect to account for repeated measurements on the same pen. For the behavioral data, a general linear mixed model (GLMM) with a gamma probability distribution and a nonlinear log-link function (state events) or a Poisson loglinear distribution (point events) compared the behaviors as a function of three fixed factors: treatment (T: C, BSF, and YM), period (P: P1, P2, and P3), week (W: W1–3 and W4–6), and their interactions. The interactions between the levels of the fixed factors were evaluated by means of pairwise comparisons. The results were expressed as the mean and standard error of the mean (SEM), and differences among treatments were considered statistically significant when the *P* values were < 0.05.

## Results

### Growth performance

The cumulative mortality rates of the C (2.38%), BSF (2.38%) and YM (4.76%) groups were not affected by the dietary treatments (*P* > 0.05). Table 4 depicts the growth performance results of the present trial. The LW, ADG, ADFI and FCR were not affected by the dietary treatments (*P* > 0.05).

**Table 4:** Growth performance of Muscovy ducks fed BSF and YM live larvae provided at 5% of the expected ADFI

**Item**	**Age**	**Dietary treatment**^a^			**SEM**	***P*-value**
		**C**	**BSF**	**YM**		
LW, g	3 d	80.7	79.9	80.4	0.599	0.890
	31 d	1,257	1,257	1,247	8.76	0.886
	55 d	2,589	2,634	2,607	11.6	0.297
ADG, g/d	3–31 d	42.0	42.0	41.7	0.301	0.876
	31–55 d	55.5	57.4	56.6	0.574	0.423
	3–55 d	48.2	49.1	48.6	0.226	0.294
ADFI (g/d) + larvae	3–31 d	81.2	79.5 + 4.54	77.2 + 4.54	0.981	0.262
	31–55 d	176	182 + 8.15	181 + 8.15	2.09	0.506
	3–55 d	125	126 + 6.21	124 + 6.21	1.20	0.772
FCR (g/g, DM basis) + larvae	3–31 d	1.76	1.75	1.72	0.025	0.859
	31–55 d	2.86	2.89	2.89	0.041	0.962
	3–55 d	2.34	2.37	2.34	0.029	0.928

*C* Control, *BSF* Black soldier fly, *YM* Yellow mealworm, *SEM* Standard error of the mean, *LW* Live weight, *ADG* Average daily gain, *ADFI* Average daily feed intake (as fed), *FCR* Feed conversion ratio (on a dry matter basis, including the larvae intake), *DM* Dry matter

^a^Three dietary treatments: C: control, commercial feed; BSF: commercial feed + 5% live BSF larvae; YM: commercial feed + 5% live YM larvae

### Larva consumption time and duck behavioral observations

**Fig. 1:**
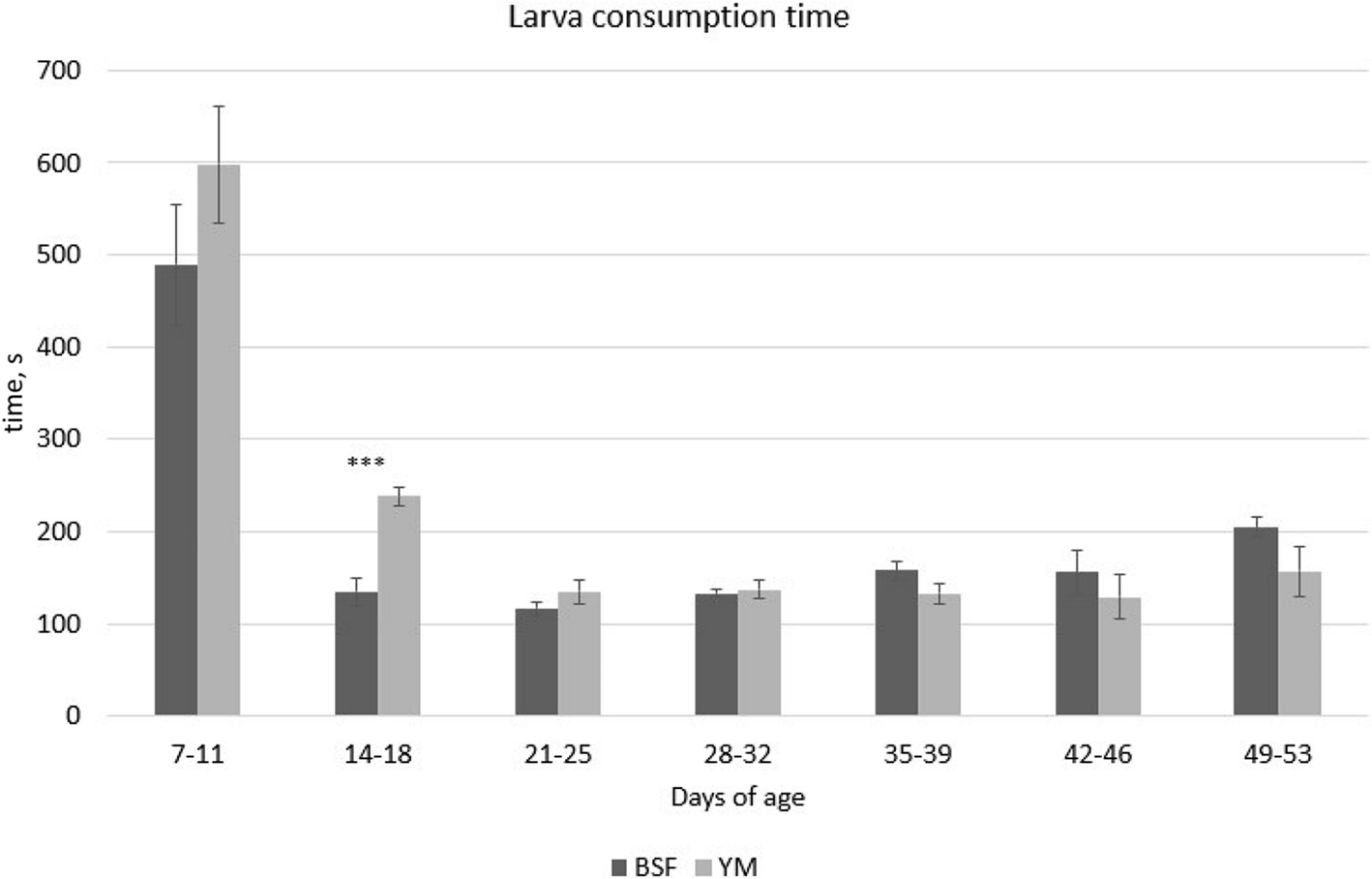
Consumption time of BSF and YM live larvae provided at 5% of the expected ADFI by the Muscovy ducks at different days of age (mean s/d interval).  BSF, black soldier fly; YM, yellow mealworm, ADFI, average daily feed intake. ^***^P < 0.001

The results of the larva consumption time are reported in Table 5 and were affected by the age of the birds, showing the longest time in larva consumption between 7–11 days of age (*P* < 0.001). The BSF and YM larva consumption times for the different ages are reported in Fig. 1. At 14–18 days of age, the birds required less time to finish consuming BSF larvae than YM larvae (*P* < 0.001). Table 6 shows the results of the observations of the state events of the Muscovy ducks. Since the interaction between T (treatment), P (period), and W (week) was not significant, the related *P*-values were not reported in Table 6. Detailed information about the interactions between the fixed effects (T, P, and W) for the state events are reported in supplementary materials (Additional file 1: Tables S1, S2, and S3). Regarding the activity behaviors, considering the overall experimental period, the insect-fed birds spent less time standing compared with the C group birds (*P* < 0.05, Table 6). By contrast, the birds of the BSF group spent more time eating and drinking compared with the C and YM groups (*P* < 0.001, Table 6). In all treatment groups, P significantly affected both activity and foraging related behaviors (*P* < 0.05, Table 6), with walking activities being highest in the hour prior to the administration of live insect larvae (P1), and standing behavior mostly performed during larva administration hour (P2). Moreover, the age of the birds significantly affected the activity behaviors independent of the treatment considered, with times spent in standing position, walking, pecking objects, and preening significantly higher during W4–6 than W1–3 (*P* < 0.05, Table 6).

**Table 5:** Larva consumption time by the Muscovy ducks

**Item**	**Treatment (T)**		**Days of age, d**							**SEM**		***P*-value**		
	**BSF**	**YM**	**7–11**	**14–18**	**21–25**	**28–32**	**35–39**	**42–46**	**49–53**	**T**	**d**	**T**	**d**	**T × d**
Larva consumption times	176	184	541^a^	180^b^	125^d^	135^c^	145^c^	142^bcd^	180^b^	11.7	15.7	0.626	<0.001	<0.001

*BSF* Black soldier fly, *YM* Yellow mealworm, *SEM* Standard error of the mean

^a–d^Means with superscript letters denote significant differences (*P* < 0.001)

**Table 6:** Behavioral observations of state events (% of total hour of observation) in Muscovy ducks fed live BSF and YM larvae provided at 5% of the expected ADFI

**Behavior category**	**Behavior**	**Treatment**^**1**^** (T)**			**Period (P)**			**Week (W)**		**SEM**			***P*-value**					
		**C**	**BSF**	**YM**	**P1**	**P2**	**P3**	**W1–3**	**W4–6**	**T**	**P**	**W**	**T**	**P**	**W**	**P × T**	**W × T**	**W × P**
Activity	Rest	64.8	61.8	63.6	62.0	64.0	64.2	69.4^α^	57.9^β^	1.96	1.96	1.60	0.539	0.679	< 0.001	0.016	0.188	0.410
	Stand	7.50^a^	4.20^b^	5.16^b^	5.47^B^	9.19^A^	3.24^C^	4.72^β^	6.33^α^	0.425	0.450	0.341	< 0.001	< 0.001	0.001	0.046	0.430	0.010
	Walk	2.84^a^	2.04^b^	2.93^a^	3.30^A^	2.51^B^	2.06^B^	2.19^β^	3.03^α^	0.231	0.233	0.189	0.007	0.001	0.002	0.247	0.510	0.247
Foraging	Eat	3.37^b^	4.63^a^	2.90^bc^	4.12^A^	3.02^B^	3.62^AB^	3.52	3.60	0.322	0.317	0.257	<0.001	0.046	0.839	0.662	0.002	0.021
	Drink	2.89^b^	4.47^a^	2.54^b^	3.79^A^	2.59^B^	3.34^A^	3.47	2.95	0.255	0.250	0.202	< 0.001	0.002	0.066	0.881	0.012	0.033
	Eat insects	-	3.00	3.36	-	3.18	-	4.27^α^	2.37^β^	0.372	0.263	0.388	0.493	-	< 0.001	-	0.092	0.493
	Peck object	2.32	2.68	2.21	2.49	2.15	2.57	2.02^β^	2.84^α^	0.281	0.281	0.230	0.495	0.535	0.012	0.292	0.157	0.288
Feather caring	Preen	15.2	18.3	16.2	18.7^A^	12.2^B^	19.9^A^	13.2^β^	20.7^α^	1.57	1.61	1.31	0.389	<0.001	< 0.001	0.001	0.480	0.210

*ADFI* Average daily feed intake, *SEM* Standard error of the mean, *C* Control, *BSF* Black soldier fly, *YM* Yellow mealworm, *P1* The hour before insect larva provision, *P2* The hour during insect larva provision, *P3* The hour after insect larva provision

^1^Three dietary treatments: C: control, commercial feed; BSF: commercial feed + 5% live BSF larvae; YM: commercial feed + 5% live YM larvae

^a–c^Means with lowercase superscript letters denote significant differences among treatments (*P* < 0.05)

^A–C^Means with uppercase superscript letters denote significant differences among periods (*P* < 0.05)

^α,β^Means with Greek alphabet superscript letters denote significant differences between week intervals (*P* < 0.05)

Some of the interactions between the fixed factors were significant. A significant P × T interaction was observed for resting, standing, and preening behaviors (*P* < 0.05, Table S1). Regarding the standing position, the YM birds spent less time standing compared to the C birds during P2, while both the insects-fed birds showed lower levels than C birds for this behavior during P3 (*P* < 0.05, Table S1). Finally, similar levels of preening behavior were observed in the birds during P1, while C and BSF birds spent more time preening during P2 than YM birds (*P* < 0.05, Table S1). By contrast, YM birds performed higher preening behavior than C birds during P3 (*P* < 0.05, Table S1). Regarding the foraging-related behaviors, W × T interaction was significant for eating and drinking (Table S2). In particular, during W4–6 the BSF birds spent more time eating and drinking than C and YM groups (*P* < 0.05, Table S2).

Table 7 reports the results of the point events behavioral observations. Detailed information about the interactions among the fixed effects (T, P, and W) of the point events are provided in supplementary materials (Additional file 1: Tables S4, S5, and S6). In general, neither the T nor the P significantly affected any of the point events evaluated (shake, stretch, flap wings, and attack); however, the occurrence of shaking movements was significantly affected by the week interval considered, being performed more frequently in W4–6 than W1–3 (*P* < 0.001, Table 7). A significant P × T interaction was revealed for stretching movements, and this behavior was mostly performed by BSF birds compared to YM during P1, and by YM birds compared to C during P3 (*P* < 0.05, Table S4). On the other hand, higher shaking movements were detected for insects-fed birds during W4–6 compared to W1–3 (*P* < 0.05, Table S5).

**Table 7:** Behavioral observations of point events (frequency % of total hour of observation) in Muscovy ducks fed live BSF and YM larvae provided at 5% of the expected ADFI

**Behavior category**	**Behavior**	**Treatment**^**1**^**(T)**			**Period (P)**			**Week (W)**		**SEM**			***P*-value**						
		**C**	**BSF**	**YM**	**P1**	**P2**	**P3**	**W** **1–3**	**W** **4–6**	**T**	**P**	**W**	**T**	**P**	**W**	**P × T**	**W × T**	**W × P**	**T × P × W**
Feather caring	Shake	0.520	0.555	0.609	0.551	0.547	0.583	0.461^β^	0.681^α^	0.036	0.036	0.030	0.226	0.750	< 0.001	0.186	0.026	0.623	0.092
	Stretch	0.079	0.101	0.091	0.098	0.081	0.092	0.090	0.091	0.008	0.008	0.007	0.171	0.322	0.908	0.011	0.194	< 0.001	0.113
	Flap wings	0.072	0.061	0.075	0.065	0.078	0.066	0.067	0.072	0.006	0.006	0.005	0.190	0.231	0.482	0.010	0.774	0.133	0.792
Aggressive	Attack	0.066	0.073	0.061	0.081	0.053	0.066	0.067	0.066	0.011	0.010	0.009	0.672	0.140	0.831	0.299	0.523	0.779	0.006

*ADFI* Average daily feed intake, *SEM* Standard error of the mean, *C* Control, *BSF* Black soldier fly, *YM* Yellow mealworm, *P1* The hour before insect larva provision, *P2* The hour during insect larva provision, *P3* The hour after insect larva provision

^1^Three dietary treatments: C: control, commercial feed; BSF: commercial feed + 5% live BSF larvae; YM: commercial feed + 5% live YM larvae

^α,β^Means with Greek alphabet superscript letters denote significant differences between week intervals (*P* < 0.05)

Regarding the frequency of attack behavior, a significant T × P × W interaction was observed (reported in Additional file 1: Fig. S1). No attacks were observed for C birds during P2 in W1–3. However, considering P1, the birds in the C group exhibited an increase in attack behavior in W4–6 with respect to W1–3 (*P* < 0.05, Fig. S1). On the contrary, no such increase was observed for BSF and YM groups. Moreover, during W1–3, the YM group showed a lower attack frequency depending on the considered P, which was the greatest during P1 and the lowest in P3 for this group (*P* < 0.05, Fig. S1).

### Excreta corticosterone metabolites

The ECM at T0 (3 days of age) and at T1 (31 days of age) were similar among the groups (Table 8). By contrast, at T2 (55 days of age) significant differences among the groups were detected, with lower ECM values observed for the BSF (−22.7%) and YM (–39.3%) groups when compared to the C group (*P* = 0.001).

**Table 8:** Excreta corticosterone metabolite (ECM) (pg/g) concentration of the Muscovy ducks fed live BSF and YM larvae provided at the 5% of the expected ADFI

**Time**	**Dietary treatment**^**1**^			**SEM**	***P*-value**
	**C**	**BSF**	**YM**		
T0	2,605	2,426	2,686	61.5	0.214
T1	3,013	3,385	3,864	17	0.132
T2	3,907^a^	3,020^b^	2,372^b^	199	0.001

*ADFI* Average daily feed intake, *C* Control, *BSF* Black soldier fly, *YM* Yellow mealworm, *SEM* Standard error of the mean, *T0* 3 days old, *T1* 31 days old, *T2* 55 days old

^1^Three dietary treatments: C: control, commercial feed; BSF: commercial feed + 5% live BSF larvae; YM: commercial feed + 5% live YM larvae

^a,b^Means with superscript letters denote significant differences among treatments (*P* < 0.05)

### Blood analyses

The blood and serum results of Muscovy ducks are reported in Table 9. At the end of the trial, the overall hematological traits were similar among the groups, except for the H/L that was affected by the dietary inclusion of live insect larvae, being lower in the insect-fed groups (on average −19.4%) compared with the C group (*P* < 0.05).

**Table 9:** Blood and serum parameters of Muscovy ducks fed live BSF and YM larvae provided at 5% of the expected ADFI

**Item**	**Dietary treatment**^**1**^			**SEM**	***P*-value**
	**C**	**BSF**	**YM**		
Erythrocytes, 10^6^ cells/μL	2.85	2.97	2.77	0.114	0.802
Leukocytes, 10^3^ cells/μL	10.2	9.61	8.36	0.569	0.418
Heterophils, %	37.7	36.4	35.5	0.898	0.627
Lymphocytes, %	56.0	58.9	59.1	0.959	0.346
Monocytes, %	2.33	2.00	2.00	0.163	0.642
Eosinophils, %	0.769	1.42	1.67	0.167	0.070
Basophils, %	2.00	1.17	1.08	0.197	0.113
H/L	0.717^a^	0.585^b^	0.573^b^	0.026	0.037
Total protein, g/dL	3.50	3.13	3.09	0.164	0.520
Triglycerides, mg/dL	51.7	64.3	49.0	2.76	0.068
Cholesterol, mg/dL	170	153	130	10.2	0.288
AST, U/L	18.2	17.0	16.7	0.983	0.802
ALT, U/L	7.91	7.82	8.08	0.359	0.956
GGT, U/L	4.17	4.50	4.50	0.224	0.789
Uric acid, mg/dL	3.12	2.95	2.69	0.154	0.538
Creatinine, mg/dL	0.078	0.077	0.074	0.003	0.810
Urea, mg/dL	1.04	1.04	0.958	0.065	0.839

*ADFI* Average daily feed intake, *C* Control, *BSF* Black soldier fly, *YM* Yellow mealworm, *SEM* Standard error of the mean, *H/L* Heterophil to lymphocyte ratio, *AST* Aspartate-aminotransferase, *ALT* Alanine-aminotransferase, *GGT* Gamma-glutamyl transferase

^1^Three dietary treatments: C: control, commercial feed; BSF: commercial feed + 5% live BSF larvae; YM: commercial feed + 5% live YM larvae

^a,b^Means with superscript letters denote significant differences among treatments (*P* < 0.05)

## Discussion

### Growth performance

The true average percentage of insect larva supplementation, as calculated at the end of the trial (according to the recorded ADFI), was 4.87% and 4.90% for BSF and YM live larvae, respectively, in line with the predicted amounts of larvae planned for the trial (5% of ADFI).

Despite the live larvae were not included in diet formulation, the nutrients supplied by the feed and larvae were similar among the groups. In the first feeding period (3–31 days of age) the total amount of nutrients was the following: 14.2 g/d of CP, 1.8 g/d of EE, 1.3 MJ/d of GE for the C group; 14.4 g/d of CP, 2.0 g/d of EE, 1.3 MJ/d of GE for the BSF group; 14.0 g/d of CP, 2.0 g/d of EE, 1.3 MJ/d of GE for the YM group. On the other hand, in the second feeding period (32–55 days of age) the total amount of nutrients was the following: 28.6 g/d of CP, 5.1 g/d of EE, 2.8 MJ/d of GE for the C group; 31.0 g/d of CP, 5.4 g/d of EE, 3.0 MJ/d of GE for the BSF group; 30.6 g/d of CP, 5.4 g/d of EE, 3.0 MJ/d of GE for the YM group. The final LW of the ducks was 9% higher compared with the rearing guidelines for this specific genotype (R61 Barred blue, Grimaud Freres Selection, France) at the same ages [35]. Our results showed that the provision of live BSF and YM larvae to Muscovy ducks did not affect the growth performance of the birds. The growth performance trends observed for the Muscovy ducks are partially in agreement with the observations of Bellezza Oddon et al. [18] for broiler chickens, where LW, ADG, and ADFI were not influenced by live BSF and YM larva provision (also provided as 5% of the expected ADFI, on an as fed basis). Partially in agreement with the present trial, Bellezza Oddon et al. [18] observed a similar overall FCR between broilers fed live BSF larvae and control birds, while they recorded a significant reduction in the overall FCR detected in broiler chickens fed live YM larvae [18]. The results of the present trial are also in line with the results reported by Bongiorno et al. [17] for Label Necked Neck chickens fed BSF live larvae provided at 10% of the expected ADFI, where, despite a higher amount of larvae provided compared with the present study, LW, ADG, ADFI and FCR were unaffected. By contrast, the daily provision of live BSF larvae (10% of the expected ADFI) to turkeys led to higher ADFI and body weight gain and a lower FCR than the control group [20]. These differences are probably related to the lower amounts of larvae provided to the animals in the present trial compared with the work by Veldkamp and van Niekerk [20] (5% vs. 10% of expected DFI of live larvae). Indeed, BSF and YM live larvae contain around 60%–70% water [36] and, for this reason, the amount of nutrients on a DM basis provided by the larvae were particularly low.

### Larva consumption time and duck behavioral observations

As far as the larva consumption time is concerned, the longer consumption times recorded at the beginning of the trial suggest that the Muscovy ducks required an amount of time to adapt to this new feed ingredient. Indeed, independent of the treatment, the Muscovy ducklings needed longer times to consume the larvae at the age of 7–11 d, approximate 544 s on average, whereas by the age of 21–25 d this time had already reduced to 125.7 s. On the other hand, comparing the two kinds of larvae provided, no difference in terms of larva consumption times were detected between BSF and YM, except for the age interval 14–18 d, where the BSF larvae were consumed 43% faster than YM larvae. Few studies have evaluated the use of live insect larvae in poultry species, and no studies had been conducted on ducks until now. A similar trend in consumption behavior was observed over time in broilers fed live BSF and YM larvae (5% of the expected ADFI) [18]. Indeed, the broiler chickens were similarly found to require an initial adaptation period to habituate to the larvae, requiring more time to complete the larva assumption during the first week compared with the following weeks of the trial. However, in contrast to what was observed in the present trial, Bellezza Oddon et al. [18] encountered different consumption times between the two insect species provided, with faster times recorded for live YM larvae compared with live BSF larvae. The results obtained in the present trial, in terms of larva consumption time, are partially in agreement with Veldkamp and van Niekerk [20], where the consumption of live BSF larvae (10% of the expected ADFI) by turkeys was completed within 2 min after provision. However, in contrast with the results in Muscovy ducks, the turkey poults did not require any adaptation period to get used to insect larva consumption [20]. These differences in larva consumption time probably vary depending on the different avian species considered and, consequently, on the differences in feeding attitudes and patterns.

Different aspects can affect the behavioral patterns of ducks, such as the time of day, the species, sex, and the presence of humans [37]. In the present work, all the birds spent most of the observation time resting (around 63.4% of the total time, on average), independent of dietary treatment, while preening was the second most frequent activity, being expressed 16.6% of the total time on average. The behavioral patterns of the present trial are mostly comparable to the behavioral patterns observed in other species of duck kept in captivity [37]. Indeed, Rose et al. [37] reported that captive ducks spend most of the time resting (42% vs. 63.4% in the present trial) and around 17% preening. They also reported that it is possible to observe differences in terms of time budget among species but in general similar trends can be observed for the most commonly behaviors.

In general, considering the overall behavioral patterns, an increase in the activity was observed according to the age of the animals. Moreover, all the birds were less active in the second hour of observation (P2) compared to P1, especially with regards to walking and drinking behavior.

Regarding the observed differences among the experimental treatments, overall, the C group spent more time in the standing position compared with BSF and YM treatments. This could mean that the control birds spent more time displaying the “alert” posture. The “alert” posture is performed standing, with the neck stretched and head erect [38], and this behavior was mainly recorded when the ducks were in the presence of human operators, who were performing larva administration in the facility.

The preening behavior increased in YM birds after larva provision, being higher than C in the considered period (P3). In ducks, preening is a complex comfort behavior, usually performed in association with shaking movements. Preening helps the animals maintain their feathers in good conditions as it helps distribute oil over the feathers from the uropygial gland [13, 38]. In the literature, an increase in preening activity is reported when ducks have access to an outdoor run or open water, and it is considered to reflect a state of well-being in ducks [13]. Thus, the increase in general preening activity in YM birds after the provision of larvae could be related to an increase in well-being in these birds compared with those of the C group.

Although the overall frequency of attack occurrences was not affected by treatment, hour of observation, or stage of duck development, a significant interaction among all these factors was found.

Contrarily to the BSF and YM groups, the frequency of attacks performed by birds in the control group increased over the weeks. This could reflect a general increase in stress experienced by these animals due to fear of humans. By contrast, in the developmental stage W1–3, the number of attacks recorded in YM birds reduced following the provision of larvae, sustaining the notion that larva provision increases well-being in ducks.

### Excreta corticosterone metabolites

At the end of the trial, in the Muscovy ducks receiving either BSF or YM supplementation the ECM concentration had significantly reduced with respect to the start of the trial. The assessment of stress response in animals is essential for the determination of their welfare since stress has a significant negative impact on animals’ immune functions and behavior [8]. The reduced ECM levels revealed in the insect-fed groups indicate lower levels of stress in these birds compared with the C group. Although the birds in the C group were exposed to the same handling procedures as the insect-fed groups (i.e., the plate presentation), a more fearful attitude was noticed in the control group birds. Indeed, unlike the control group birds, the insect-supplemented groups tended to approach the operators at the time of larva provision and appeared less fearful towards the operators in general. The positive association between the appearance of operators in the facility and the provision of larvae probably played a key role in stress reduction in the treated animals.

### Blood analyses

As a whole, the hematological and serum parameters were not affected by the treatments, with the exception of the H/L ratio, for which lower values were observed in the insect-supplemented groups compared with the C birds. The latter finding contrasts with what was observed in broiler chickens fed BSF and YM live larvae (5% of the expected ADFI), as the H/L was unaffected by the experimental treatments in broilers [18]. The H/L has been reported to be a sensitive hematological indicator of stress in poultry, which increases in the case of environmental stressors or conditions of reduced welfare [10, 11]. The correlation between the ECM concentration and the H/L in vertebrates and in birds is not very clear since the interaction between these two parameters is highly complex, being influenced by many factors [39]. A study conducted by Alm et al. [40] on laying hens reported a weak correlation between H/L and ECM. Thus, the correlation between ECM and H/L was also investigated in the present trial, revealing a positive, but non-significant, correlation between the two (Spearman correlation coefficient, *r*_s_ = 0.252; *P* = 0.313).

## Conclusions

The growth performance of the animals was not significantly affected by the provision of larvae. The results on dietary supplementation with live BSF and YM larvae were promising in terms of improving the welfare of captivity-farmed Muscovy ducks as it reduced the ECM and H/L in insect-fed birds compared to C birds. The absence of differences in terms of larva consumption times between BSF and YM birds seems to suggest that no preferences between the two insect species provided occurred. The reduction in attack frequency observed for YM birds during P3 in the first developmental stage (W1–3) may be related to the positive stimuli of live YM larva provision. Moreover, in YM birds also the preening behavior was improved by the larva provision, reflecting an increase on these animals’ well-being. On the contrary, the C birds displayed higher “alert” posture (i.e., standing behavior) compared to insects-fed birds, resulting in a more fearful attitude towards the operators.

Since abnormal and aggressive behaviors are one of the most difficult problems to solve in duck rearing, the possibility of reducing these phenomena by supplementing the ducks’ diet with insects should be investigated further to increase the birds’ welfare in this type of production system.

### Supplementary Information

**Additional file 1: Table S1.** Effect of period × treatment (P × T) on the behavioral observations of state events in Muscovy ducks fed live BSF and YM larvae provided at 5% of the expected ADFI. **Table S2.** Effect of week × treatment (W × T) on the behavioral observations of state events in Muscovy ducks fed live BSF and YM larvae provided at 5% of the expected ADFI. **Table S3.** Effect of week × period (W × P) on the behavioral observations of state events in Muscovy ducks fed live BSF and YM larvae provided at 5% of the expected ADFI. **Table S4.** Effect of period × treatment (P × T) on the behavioral observations of point events in Muscovy ducks fed live BSF and YM larvae provided at 5% of the expected ADFI. **Table S5.** Effect of week × treatment (W × T) on the behavioral observations of point events in Muscovy ducks fed live BSF and YM larvae provided at 5% of the expected ADFI. **Table S6.** Effect of week × period (W × P) on the behavioral observations of point events in Muscovy ducks fed live BSF and YM larvae provided at 5% of the expected ADFI. **Fig. S1.** Effect of treatment × period × week (T × P × W) on the attack behavior in Muscovy ducks fed live BSF and YM larvae provided at 5% of the expected ADFI.

## Data Availability

The datasets used and/or analyzed during the current study are available from the corresponding author on reasonable request.

## Abbreviations

ADFI: Average daily feed intake
ADG: Average daily gain
ALT: Alanine-aminotransferase
AMEn: Apparent metabolizable energy corrected for nitrogen
AST: Aspartate-aminotransferase
BSF: Black soldier fly
CP: Crude protein
DM: Dry matter
ECM: Excreta corticosterone metabolites
EE: Ether extract
FCR: Feed conversion ratio
GE: Gross energy
GGLM: General linear mixed model
GGT: Gamma glutamyl transferase
H/L: Heterophil to lymphocyte ratio
LW: Live weight
P: Periods
SEM: Standard error of the mean
T: Treatment
YM: Yellow mealworm
