# Supplementary material for: Does the provision of live black soldier fly and yellow mealworm larvae improve Muscovy duck welfare?
Source: J Anim Sci Biotechnol. 2023 Dec 4;14:153. doi: 10.1186/s40104-023-00949-7 (PMC10694867; doi:10.1186/s40104-023-00949-7)
Supplement: Supplementary file 1 — Additional file 1: Table S1. Effect of period × treatment (P × T) on the behavioral observations of state events in Muscovy ducks fed live BSF and YM larvae provided at 5% of the expected ADFI. Table S2. Effect of week × treatment (W × T) on the behavioral observations of state events in Muscovy ducks fed live BSF and YM larvae provided at 5% of the expected ADFI. Table S3. Effect of week × period (W × P) on the behavioral observations of state events in Muscovy ducks fed live BSF and YM larvae provided at 5% of the expected ADFI. Table S4. Effect of period × treatment (P × T) on the behavioral observations of point events in Muscovy ducks fed live BSF and YM larvae provided at 5% of the expected ADFI. Table S5. Effect of week × treatment (W × T) on the behavioral observations of point events in Muscovy ducks fed live BSF and YM larvae provided at 5% of the expected ADFI. Table S6. Effect of week × period (W × P) on the behavioral observations of point events in Muscovy ducks fed live BSF and YM larvae provided at 5% of the expected ADFI. Fig. S1. Effect of treatment × period × week (T × P × W) on the attack behavior in Muscovy ducks fed live BSF and YM larvae provided at 5% of the expected ADFI. [file 40104_2023_949_MOESM1_ESM.docx]

**Additional file 1**

**Table S1** Effect of period × treatment (P × T) on the behavioral observations of state events in Muscovy ducks fed live BSF and YM larvae provided at 5% of the expected ADFI

| **Behavior category** | **Behavior** | **P1** | | | **P2** | | | **P3** | | | **SEM** | ***P*-value** |
| --- | --- | --- | --- | --- | --- | --- | --- | --- | --- | --- | --- | --- |
|  |  | **C** | **BSF** | **YM** | **C** | **BSF** | **YM** | **C** | **BSF** | **YM** | **P × T** |  |
| Activity | Rest | 63.0 | 62.6^x^ | 60.4^y^ | 64.2^ab^ | 56.9^b,xy^ | 71.9^a,x^ | 67.4 | 66.3^x^ | 59.2^y^ | 3.40 | 0.016 |
|  | Stand | 6.45^a,y^ | 4.44^b,y^ | 5.73^ab,xy^ | 12.13^a,x^ | 8.45^ab,x^ | 7.57^b,y^ | 5.38^a,y^ | 2.01^c,z^ | 3.16^b,z^ | 0.800 | 0.046 |
|  | Walk | 3.76 | 2.56 | 3.74 | 2.31 | 2.44 | 2.79 | 2.64 | 1.37 | 2.41 | 0.409 | 0.247 |
| Foraging | Eat | 3.43 | 5.40 | 3.79 | 2.95 | 4.13 | 2.27 | 3.78 | 4.44 | 2.83 | 0.563 | 0.662 |
|  | Drink | 3.15 | 5.42 | 3.20 | 2.46 | 3.69 | 1.92 | 3.10 | 4.49 | 2.67 | 0.448 | 0.881 |
|  | Eat Insect | - | - | - | - | 3.00 | 3.37 | - | - | - | 0.372 | - |
|  | Peck Object | 2.22 | 3.36 | 2.08 | 1.82 | 2.58 | 2.14 | 3.11 | 2.23 | 2.46 | 0.490 | 0.292 |
| Feather caring | Preen | 19.1 | 16.9 | 20.3^x^ | 13.3^a^ | 17.1^a^ | 7.94^b,y^ | 13.9^b^ | 21.1^ab^ | 26.6^a,x^ | 2.85 | 0.001 |

*ADFI* Average daily feed intake, *P1* The hour before insect larvae provision, *P2* The hour during insect larvae provision, *P3* The hour after insect larvae provision, *SEM* Standard error of the mean, *C* Control, *BSF* Black soldier fly, *YM* Yellow mealworm, *T* Treatment, *P* Period

^a–c^Means with superscript letters denote significant differences among treatments (C, BSF, and YM) in the considered period (P1, P2 or P3) (*P* < 0.05)

^x–z^Means with superscript letters denote significant differences of the same treatment among the periods (P1, P2 and P3) (*P* < 0.05)

**Table S2** Effect of week × treatment (W × T) on the behavioral observations of state events in Muscovy ducks fed live BSF and YM larvae provided at 5% of the expected ADFI

| **Behavior category** | **Behavior** | **W1–3** | | | **W4–6** | | | **SEM** | ***P*-value** |
| --- | --- | --- | --- | --- | --- | --- | --- | --- | --- |
|  |  | **C** | **BSF** | **YM** | **C** | **BSF** | **YM** | **W × T** |  |
| Activity | Rest | 67.8 | 69.6 | 70.7 | 62.0 | 54.8 | 57.2 | 2.78 | 0.188 |
|  | Stand | 6.65 | 3.37 | 4.70 | 8.46 | 5.29 | 5.66 | 0.606 | 0.430 |
|  | Walk | 2.45 | 1.85 | 2.30 | 3.30 | 2.26 | 3.74 | 0.332 | 0.510 |
| Foraging | Eat | 4.07^x^ | 3.60^y^ | 2.99 | 2.79^b,y^ | 5.95^a,x^ | 2.81^b^ | 0.463 | 0.002 |
|  | Drink | 3.76^a,x^ | 4.35^a^ | 2.57^b^ | 2.22^b,y^ | 4.61^a^ | 2.52^b^ | 0.364 | 0.012 |
|  | Eat Insect | - | 3.51 | 5.19 | - | 2.57 | 2.18 | 0.556 | 0.092 |
|  | Peck Object | 2.19 | 1.89 | 2.01 | 2.47 | 3.80 | 2.45 | 0.402 | 0.157 |
| Feather caring | Preen | 12.8 | 15.2 | 11.8 | 18.0 | 22.0 | 22.3 | 2.28 | 0.480 |

*ADFI* Average daily feed intake, *W* Week, *SEM* Standard error of the mean, *C* Control, *BSF* Black soldier fly, *YM* Yellow mealworm, *T* Treatment

Three dietary treatments: C: control, commercial feed; BSF: commercial feed + 5% live BSF larvae; YM: commercial feed + 5% live YM larvae

^a,b^Means with superscript letters denote significant differences among treatments (C, BSF, and YM) in the considered week interval (W1–3 or W4–6) (*P* < 0.05)

^x,y^Means with superscript letters denote significant differences of the same treatment between the week intervals (W1–3 and W4–6) (*P* < 0.05)

**Table S3** Effect of week × period (W × P) on the behavioral observations of state events in Muscovy ducks fed live BSF and YM larvae provided at 5% of the expected ADFI

| **Behavior category** | **Behavior** | **W1–3** | | | **W4–6** | | | **SEM** | ***P*-value** |
| --- | --- | --- | --- | --- | --- | --- | --- | --- | --- |
|  |  | **P1** | **P2** | **P3** | **P1** | **P2** | **P3** | **W × P** |  |
| Activity | Rest | 65.6 | 71.5 | 71.1 | 58.6 | 57.3 | 57.9 | 2.78 | 0.410 |
|  | Stand | 5.49^a^ | 6.70^a,y^ | 2.86^b^ | 5.45^b^ | 12.6^a,x^ | 3.68^c^ | 0.653 | 0.010 |
|  | Walk | 3.19 | 1.94 | 1.69 | 3.42 | 3.24 | 2.51 | 0.334 | 0.247 |
| Foraging | Eat | 3.90^a^ | 2.59^b^ | 4.34^a,x^ | 4.37^a^ | 3.54^ab^ | 3.02^b,y^ | 0.453 | 0.021 |
|  | Drink | 3.52 | 2.91 | 4.10^x^ | 4.09^a^ | 2.32^b^ | 2.72^b,y^ | 0.358 | 0.033 |
|  | Eat Insects | - | 4.27 | - | - | 2.37 | - | 0.388 | 0.493 |
|  | Peck Object | 1.89 | 2.11 | 2.08 | 3.30 | 2.20 | 3.18 | 0.400 | 0.288 |
| Feather caring | Preen | 17.1 | 9.29 | 14.5 | 20.4 | 15.9 | 27.1 | 2.34 | 0.210 |

*ADFI* Average daily feed intake, *W* Week, *SEM* Standard error of the mean, *P1* The hour before insect larvae provision, *P2* The hour during insect larvae provision, *P3* The hour after insect larvae provision, *P* Period

^a–c^Means with superscript letters denote significant differences among periods (P1, P2, and P3) in the considered week interval (W1–3 or W4–6) (*P* < 0.05)

^x,y^Means with superscript letters denote significant differences of the same period between the week intervals (W1–3 and W4–6) (*P* < 0.05)

**Table S4** Effect of period × treatment (P × T) on the behavioral observations of point events in Muscovy ducks fed live BSF and YM larvae provided at 5% of the expected ADFI

| **Behavior category** | **Behavior** | **P1** | | | **P2** | | | **P3** | | | **SEM** | ***P*-value** |
| --- | --- | --- | --- | --- | --- | --- | --- | --- | --- | --- | --- | --- |
|  |  | **C** | **BSF** | **YM** | **C** | **BSF** | **YM** | **C** | **BSF** | **YM** | **P × T** |  |
| Feather caring | Shake | 0.522 | 0.492 | 0.652 | 0.508 | 0.636 | 0.508 | 0.532 | 0.546 | 0.682 | 0.063 | 0.186 |
|  | Stretch | 0.108^ab,x^ | 0.127^a^ | 0.069^b,y^ | 0.062^y^ | 0.087 | 0.098^xy^ | 0.073^b,xy^ | 0.093^ab^ | 0.113^a,x^ | 0.014 | 0.011 |
|  | Flap wings | 0.082 | 0.056^y^ | 0.060^y^ | 0.075 | 0.084^x^ | 0.074^xy^ | 0.061^b^ | 0.049^b,y^ | 0.095^a,x^ | 0.010 | 0.010 |
| Aggressive | Attack | 0.062 | 0.096 | 0.089 | 0.045 | 0.054 | 0.058 | 0.085 | 0.077 | 0.044 | 0.019 | 0.299 |

*ADFI* Average daily feed intake, *P1* The hour before insect larvae provision, *P2* The hour during insect larvae provision, *P3* The hour after insect larvae provision, *SEM* Standard error of the mean, *C* Control, *BSF* Black soldier fly, *YM* Yellow mealworm, *T* Treatment, *P* Period

^a,b^Means with superscript letters denote significant differences between treatments (C, BSF, and YM) in the considered period (P1, P2 or P3) (*P* < 0.05)

^x,y^Means with superscript letters denote significant differences of the same treatment among periods (P1, P2 and P3) (*P* < 0.05)

**Table S5** Effect of week × treatment (W × T) on the behavioral observations of point events in Muscovy ducks fed live BSF and YM larvae provided at 5% of the expected ADFI

| **Behavior category** | **Behavior** | **W1–3** | | | **W4–6** | | | **SEM** | ***P*-value** |
| --- | --- | --- | --- | --- | --- | --- | --- | --- | --- |
|  |  | **C** | **BSF** | **YM** | **C** | **BSF** | **YM** | **W × T** |  |
| Feather caring | Shake | 0.480 | 0.400^y^ | 0.511^y^ | 0.565^b^ | 0.769^a,x^ | 0.726^ab,x^ | 0.053 | 0.026 |
|  | Stretch | 0.071 | 0.113 | 0.090 | 0.089 | 0.090 | 0.093 | 0.012 | 0.194 |
|  | Flap wings | 0.073 | 0.058 | 0.071 | 0.071 | 0.065 | 0.079 | 0.008 | 0.774 |
| Aggressive | Attack | 0.057 | 0.076 | 0.066 | 0.072 | 0.071 | 0.056 | 0.015 | 0.523 |

*ADFI* Average daily feed intake, *W* Week, *SEM* Standard error of the mean, *C* Control, *BSF* Black soldier fly, *YM* Yellow mealworm, *T* Treatment

Three dietary treatments: C: control, commercial feed; BSF: commercial feed + 5% live BSF larvae; YM: commercial feed + 5% live YM larvae

^a,b^Means with superscript letters denote significant differences among treatments (C, BSF, and YM) in the considered week interval (W1–3 or W4–6) (*P* < 0.05)

^x,y^Means with superscript letters denote significant differences of the same treatment between the week intervals (W1–3 and W4–6) (*P* < 0.05)

**Table S6** Effect of week × period (W × P) on the behavioral observations of point events in Muscovy ducks fed live BSF and YM larvae provided at 5% of the expected ADFI

| **Behavior category** | **Behavior** | **W1–3** | | | **W4–6** | | | **SEM** | ***P*-value** |
| --- | --- | --- | --- | --- | --- | --- | --- | --- | --- |
|  |  | **P1** | **P2** | **P3** | **P1** | **P2** | **P3** | **W × P** |  |
| Feather caring | Shake | 0.440 | 0.441 | 0.505 | 0.689 | 0.679 | 0.673 | 0.052 | 0.623 |
|  | Stretch | 0.092^ab^ | 0.063^b,y^ | 0.124^a,x^ | 0.105^a^ | 0.104^a,x^ | 0.068^b,y^ | 0.012 | 0.000 |
|  | Flap wings | 0.055 | 0.082 | 0.067 | 0.077 | 0.074 | 0.065 | 0.008 | 0.133 |
| Aggressive | Attack | 0.073 | 0.065 | 0.064 | 0.090 | 0.047 | 0.067 | 0.015 | 0.779 |

*ADFI* Average daily feed intake, *W* Week, *SEM* Standard error of the mean, *P1* The hour before insect larvae provision, *P2* The hour during insect larvae provision, *P3* The hour after insect larvae provision, *P* Period

^a,b^Means with superscript letters denote significant differences among periods (P1, P2, and P3) in the considered week interval (W1–3 or W4–6) (*P* < 0.05)

^x,y^Means with superscript letters denote significant differences of the same period between the week intervals (W1–3 and W4–6) (*P* < 0.05)


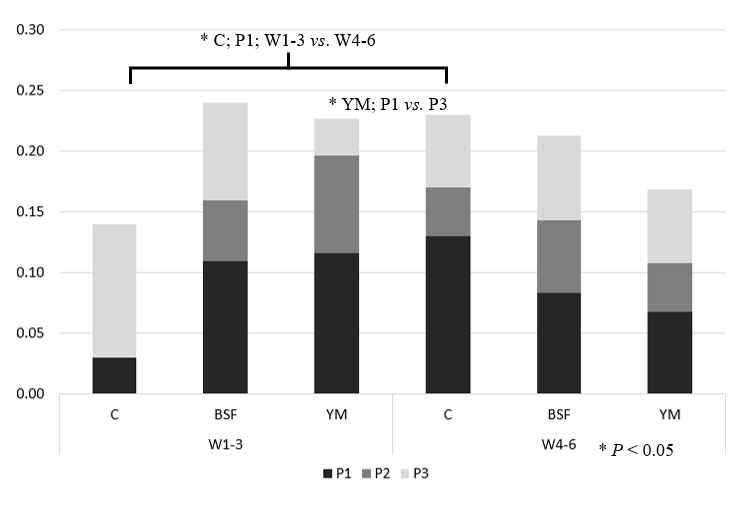


**Fig. S1** Effect of treatment × period × week (T × P × W) on the attack behavior in Muscovy ducks fed live BSF and YM larvae provided at 5% of the expected ADFI. ADFI, average daily feed intake; P1, the hour before insect larvae provision; P2, the hour during insect larvae provision; P3, the hour after insect larvae provision; C, control; BSF, black soldier fly; YM, yellow mealworm; T, treatment; P, period; W, week
